# Prospective Cohort Study in Alport Syndrome Patients Under Standard Therapy

**DOI:** 10.1016/j.ekir.2025.02.036

**Published:** 2025-03-06

**Authors:** Oliver Gross, Michelle N. Rheault, James Simon, Bertrand Knebelmann, Yuqian Shen, Qi Zhang, Ali Hariri, Julie Lin, Shiguang Liu, Clifford E. Kashtan

**Affiliations:** 1 University Medical Center Goettingen, Nephrology and Rheumatology, Goettingen, Germany; 2 University of Minnesota Masonic Children’s Hospital, Minneapolis, Minnesota, USA; 3 Cleveland Clinic, Cleveland, Ohio, USA; 4 Necker Hospital, APHP, Université Paris Cité, Paris, France; 5 Sanofi, Cambridge, Massachusetts, USA

**Keywords:** albuminuria, Alport syndrome, biomarkers, chronic kidney disease, clinical trials, kidney disease progression

## Abstract

**Introduction:**

Patients with Alport syndrome (AS), a common genetic kidney disease, exhibit variable rates of decline in kidney function. Consequently, this global, multicenter, prospective observational study aimed to generate data useful for designing future interventional trials.

**Methods:**

The study included patients aged 12 to 65 years with a confirmed diagnosis of AS and estimated glomerular filtration rate (eGFR) of 45 to 90 ml/min. For up to 120 weeks in 12-weekly intervals, blood and urine samples, patient and family history, genotype, adverse events (AEs), medications, and patient-related outcome data were collected under International Conference on Harmonization-Good Clinical Practice (ICH-GCP) standards.

**Results:**

Out of 165 patients enrolled, 101 (61.2%) were classified as X-linked (62.4% females, 37.6% males) and 32 (19.4%) as autosomal (recessive or dominant) inheritance. Baseline mean eGFR was 64 ml/min per 1.73 m^2^, and yearly eGFR slope in ml/min per 1.73 m^2^ was −2.94 (−6.7 in X-linked males, 0.6 in X-linked females, −1.66 in heterozygous autosomal patients). Baseline urine albumin-to-creatinine ratio (UACR) was the best predictor for rapid loss of eGFR with a yearly eGFR slope of −10.16 ml/min per 1.73 m^2^ in patients with UACR > 1 g/g compared with−0.90 ml/min per 1.73 m^2^ if UACR was ≤ 1.0 g/g. Out of 353 AEs, only 26 (7.4%) were related to AS. In addition to UACR, neutrophil gelatinase-associated lipocalin, clusterin, and kidney injury molecule-1 correlated with the eGFR slope.

**Conclusion:**

In patients with AS receiving standard of care, rapid decline in kidney function strongly correlates with UACR and AEs related to the underlying medical condition are rare. Both findings enrich the design of future interventional trials.

## Graphical abstract

**Figure:**
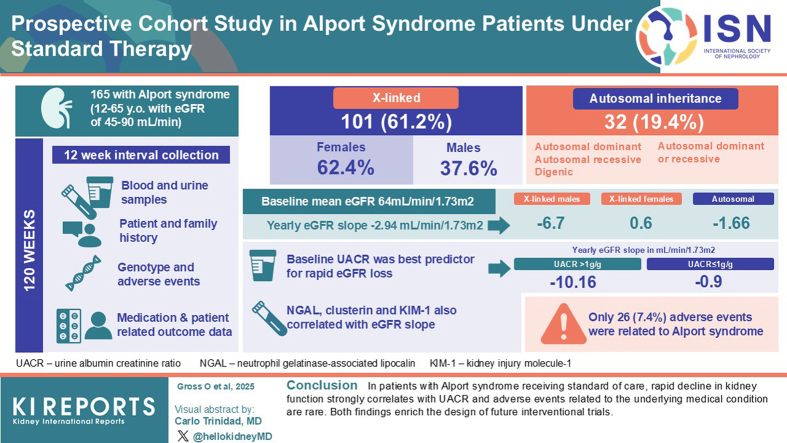

AS is a common hereditary glomerular kidney disease. As a disease of type IV collagen caused by variants in collagen IV genes (COL4A3, COL4A4, and COL4A5), AS weakens the structural integrity of basement membranes, and leads to chronic kidney disease (CKD), hearing loss, and eye abnormalities.^1^^,^^2^

Variants in *COL4A5* cause X-linked AS (XLAS). Males with XLAS (hemizygous XLAS) manifest hematuria at a very young age, followed by proteinuria, and decline in eGFR.^3^^,^^4^ If untreated, most males with XLAS will develop end-stage kidney failure (ESKF) and require kidney replacement therapy by the age of 25 years; large deletions, nonsense variants, or variants changing the reading frame are associated with a higher risk of early-onset ESKF and poorer response to therapy than missense variants.^3^, ^4^, ^5^ Blockade of the renin-angiotensin system (RAS), preferably with the angiotensin-converting enzyme inhibitor, ramipril, has been shown to delay ESKF, especially if started early in the course of disease.^4^, ^5^, ^6^ Heterozygous women with XLAS show widely variable kidney involvement from isolated lifelong hematuria without progressive CKD to overt proteinuria, CKD, or ESKF.^7^, ^8^, ^9^ If untreated, 30% to 40% of women with XLAS reach ESKF by age 60 years.^7^ Lifelong risk for ESKF in women with XLAS can be reduced from 30% to 40% to < 5% by early nephroprotective RAS-blockade.^9^^,^^10^ Individuals with autosomal recessive AS follow a pattern of kidney disease progression similar to males with XLAS and show a similar genotype-response to therapy correlation.^2^^,^^11^ ESKF can also be delayed by years, again depending on the genotype and timing of therapy.^11^ Autosomal dominant inheritance pattern is seen in up to 30% of patients with AS. These patients have a widely variable presentation, with rare and much less rapid progression of kidney disease; progression of CKD may be halted by timely RAS-blockade in most cases, depending on additional risk factors such as smoking.^10^

Timely RAS-blockade, before onset of kidney failure, delays ESKF by a median of 18 years and has been shown to prolong kidney survival and life expectancy in patients with AS in prospective and retrospective observational studies.^4^, ^5^, ^6^^,^^9^^,^^10^^,^^12^^,^^13^ The first randomized controlled clinical trial in AS, EARLY PRO-TECT Alport, a multicenter, placebo-controlled, double-blind phase 3 trial with open-arm comparison, indicated safety and efficacy of nephroprotective therapy with ramipril in children with AS.^6^ Data from EARLY PRO-TECT Alport suggest that starting RAS-blockade with the angiotensin-converting enzyme inhibitor, ramipril, before onset of microalbuminuria can be beneficial in all patients 2 years and older with hemizygous XLAS or autosomal recessive AS regardless of the presence of albuminuria and with onset of microalbuminuria in all other patients.^4^, ^5^, ^6^^,^^9^^,^^10^^,^^12^^,^^13^

Nephroprotective effects of early RAS-blockade, however, are not a cure for patients with AS. Many patients still progress to ESKF early in life. Consequently, there is an urgent need for therapeutical trials investigating therapies added to RAS-blockade in AS.^14^ Given the multiple inheritance patterns of patients with AS and the different responses to RAS-blockade, which partly depends on the biochemical effects and altered cellular trafficking of the type IV collagen protein resulting from different gene-variants, it is challenging to identify patients with AS who are at risk of faster progression of their kidney disease. Little is known about the rate of decline of established markers of kidney function, such as glomerular filtration rate (GFR). Identification of the patients who progress more rapidly (fast progressors) in AS is of special interest because enrolling such patients in clinical treatment trials is essential to showing potential treatment benefits of study medications. Therefore, we conducted the first prospective, global, multicenter natural history study of patients with AS under standard of care, to characterize the natural decline of kidney function, AEs, and to identify novel predictors of progressive kidney disease that may be useful in the design of future clinical trials.

## Methods

ATHENA: Natural History of Disease Study in Alport Syndrome Patients (NCT02136862, registered May 13, 2014) was a global, multicenter, prospective observational study originally sponsored by Regulus Therapeutics before being transferred to Genzyme, a Sanofi Company. Enrollment began in September 2014 and concluded in December 2017; 16 sites in 7 countries (Australia, Canada, France, Germany, Spain, the United Kingdom, and the United States) were included.

The study’s primary objective was to characterize the change in established kidney-function markers (GFR, UACR, and creatinine) in participants with AS for up to 120 weeks. The secondary objectives were as follows: (i) to prospectively document AEs under high-quality ICH-GCP conditions, including immediate reporting of severe AEs (SAEs) as in phase 2 or phase 3 clinical trials; (ii) to evaluate correlations between kidney disease progression and exploratory biomarkers in blood and urine; and (iii) to investigate correlations between the rate of kidney disease progression and genotype. The full list of inclusion and exclusion criteria is provided in the Supplementary Methods.

Baseline and demographic information obtained during the baseline visit included past medical history; family history of AS; treatment history; concomitant medications; body system assessment (targeted to body systems of interest with questions about recent illness); body weight and height; vital signs; urine samples (first-void and 24-h); and blood samples for clinical chemistry, hematology, coagulation, biomarkers and genetic testing for the *COL4A3, COL4A4*, and *COL4A5* genes. Genetic testing was performed by Machaon Diagnostics (Oakland, CA) in a Clinical Laboratory Improvement Amendments–approved laboratory. The genetic variants were annotated using the current databases, literature, and variant interpretation guidelines from the American College of Medical Genetics and Genomics; further information on genetic sequencing can be found in the Supplementary Methods.^15^, ^16^, ^17^, ^18^ Body system assessment, vital signs, and blood and urine sample collection were performed every 12 weeks for up to 120 weeks.

AEs (excluding preexisting conditions) and SAEs were recorded in the case report form, including the description of the AE, the date and time of both onset and resolution, severity, causality, action taken, and outcome. AEs were followed-up with until the condition had stabilized and the investigator felt no further follow-up was warranted.

Measured GFR performed with iohexol clearance and eGFR calculated using the Chronic Kidney Disease Epidemiology Collaboration creatinine-cystatin C equation were assessed in participants enrolled before implementation of protocol amendment 3. However, because of close correlation between the 2 variables during this period, only eGFR was used after protocol amendment 3. eGFR was assessed every 24 weeks until week 96, plus at the end of study at week 120 or early-termination visit. Urine biomarkers were adjusted for urinary creatinine to account for variability in urine concentration. Central laboratory assessments were performed by LabConnect in the United States, Synevo Central Labs in the European Union, or Sonic Clinical Trials in Australia. Further detail on exploratory urine and blood biomarker assessments can be found in the Supplementary Methods. Quality-of-life assessments (including SF-36) were administered at baseline and at 6-month intervals; these results will be reported on separately.

### Statistical Methods

In general, continuous data were summarized using the number of observations available: mean and (SD [median, range]). Categorical and ordinal data were summarized using the count and percentage of participants. Subgroup analyses in eGFR slope, as well as demographic and baseline disease characteristics, by key biomarkers’ severity level and likely genotype inheritance were performed.

A median plot of eGFR over the course of the study by genotype subgroup was created. For participants with ≥ 2 eGFR datapoints collected during the study, eGFR slopes were calculated using linear regression. eGFR slopes were summarized by subgroup, including male or female sex, genotype, and level of proteinuria or albuminuria. As a *posthoc* analysis, Pearson correlations between eGFR slope and baseline urine biomarker values, including 24-hour urine protein, urine protein-to-creatinine ratio (UPCR), and UACR were calculated. Partial Pearson correlation coefficients adjusted by participant effect were performed to assess correlation between eGFR and biomarkers over time during the study. All biomarkers collected in urine were standardized by calculating the ratio between urine parameters and creatinine; in addition, logarithmic values were used as needed to improve data normality. Further detail on *posthoc* analyses (including a multivariable logistic regression to identify the predictors for rapid eGFR progression, defined as eGFR slope ≤ −4 ml/min per 1.73 m^2^ per year) can be found in the Supplementary Materials. All analyses were performed in SAS version 9.4 (SAS Institute Inc., Cary, North Carolina, USA).

## Results

**Figure 1:**
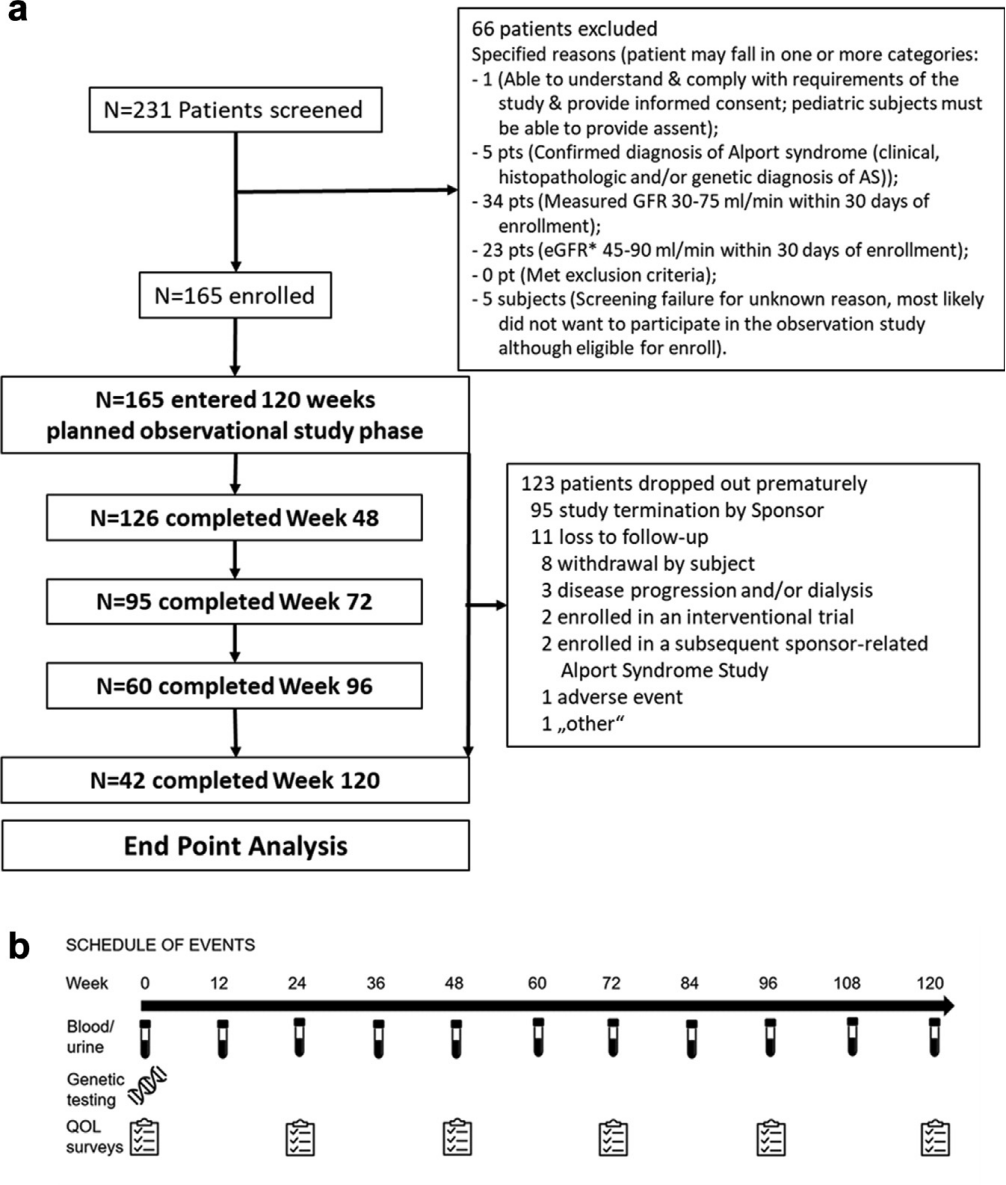
ATHENA study STROBE diagram and schedule of events. QOL, quality of life.

A total of 165 participants with AS were screened and enrolled in the ATHENA study (Figure 1), making ATHENA the world’s largest observational ICH-GCP–conforming study ever done in AS. More than a quarter of the patients (*n* = 42; 25.5%) completed the study through week 120 (end-of-study visit) and 95 participated until termination of the study by Regulus Therapeutics (for strategic, nonclinical reasons) before week 120. The duration of median (interquartile range) follow-up was 90 (66–120) weeks. A total of 28 patients (17.0%) dropped out prematurely for the following reasons other than “study termination by sponsor”: lost to follow-up (*n* = 11, 6.7%), withdrawal by participant (*n* = 8, 4.8%), disease progression and/or dialysis (*n* = 3, 1.8%), enrollment in interventional trial or received kidney transplant (*n* = 4, 2.4%), 1 AE (ESKF), and 1 (patient is no longer available for study visits and does not want to continue).

Baseline demographic and clinical characteristics are shown in Table 1. Most participants (98%) received concomitant medications during the study period; most commonly RAAS blockade (81%), including angiotensin-converting enzyme inhibitor (35%), angiotensin receptor blocker (30%), or both (16%). Most of the 165 patients were female (*n* = 109, 66%). XLAS was the most common form of inheritance (61.2%), including 57.8% of females (*n* = 63) and 67.9% of males (*n* = 38). Autosomal AS (dominant or compound heterozygous with 1 pathogenic or likely pathogenic plus 1 variant of unknown significance) was the second most common form of inheritance (18.2%), followed by autosomal recessive (*n* = 2) and digenic (*n* = 2) forms of AS (Table 2). Of note, as shown in Supplementary Table S1, several females in the autosomal dominant AS group had additional autosomal variants of unknown significance, which were, from a purely genetic perspective, not classified as pathogenic despite kidney biopsies confirmative for (autosomal recessive) AS. For that reason, the complete American College of Medical Genetics and Genomics classification is shown in Supplementary Table S1.

**Table 1:** Baseline demographics and clinical information for all enrolled participants

Baseline measures	All enrolled participants (*N* = 165)
Age (yrs)	
Mean (SD)	45 (14)
Median (range)	46 (15–80)
Sex, *n* (%)	
Female	109 (66)
Male	56 (34)
Race/ethnicity, *n* (%)	
Asian	7 (4)
Black or African American	1 (0.6)
Other^a^/not reported	20 (12)
White	137 (83)
eGFR (CKD-EPI 2009) (ml/min per 1.73 m^2^)	
Mean (SD)	64 (22)
Median (range)	63 (18–124)
Urine albumin-to-creatinine ratio (g/g)	
Mean (SD)	0.600 (0.800)
Median (range)	0.200 (0–6.000)
Urine protein (mg/24 h)	
Mean (SD)	1844 (2608)
Median (range)	684 (13–14,073)
Diastolic blood pressure (mm Hg)	
Mean (SD)	75 (11)
Median	74 (48–106)
Systolic blood pressure (mm Hg)	
Mean (SD)	126 (17)
Median (range)	124 (87–186)
Body mass index (BMI)	
Mean (SD)	26.76 (5.714)
Median (range)	26.04 (17.8–51.1)

	Female (109)^b^	Male (56)
X-linked inheritance	63	38
Missense variant	40	31
Nonmissense variant (all others)	23	7
Autosomal (heterozygous)^c^	24	6
Autosomal recessive^d^	2	0
Missing data or no mutation identified	16	6

BMI, body mass index; CKD-EPI, Chronic Kidney Disease Epidemiology Collaboration equation; eGFR, estimated glomerular filtration rate; VUS, variant of unknown significance.

a Race Other includes participants from France and those who selected multiple race categories.

b Four females (1 digenic and 3 recessive or dominant) were not presented in the genetic analysis.

c Dominant or other variant classified as VUS.

d Only patients with 2 pathogenic or likely pathogenic autosomal variants included here, autosomal compound heterozygous patients excluded with 1 likely pathogenic or pathogenic variant plus 1 variant of unknown significance.

**Table 2:** Summary of likely inheritance and type of mutation for the most pathogenic variant–all enrolled participants

Mode of inheritance, *n* (%)	Type of mutation for the most pathogenic variant	Female (*n* = 109)	Male (*n* = 56)	Overall (*N* = 165)
X-linked	Missense	40 (64)	31 (82)	71 (70)
	Frameshift	5 (8)	3 (8)	8 (8)
	Splice site	6 (10)	1 (3)	7 (7)
	Nonsense	4 (6)	2 (5)	6 (6)
	Noncoding	4 (6)	1 (3)	5 (5)
	Nonframeshift	2 (3)	0	2 (2)
	Synonymous	2 (3)	0	2 (2)
Autosomal recessive	Missense, nonframeshift	1 (50)	0	1 (50)
	Splice site	1 (50)	0	1 (50)
Autosomal dominant	Missense	14 (58)	2 (33)	16 (53)
	Splice site	3 (13)	2 (33)	5 (17)
	Noncoding	1 (4)	2 (33)	3 (10)
	Frameshift	2 (8)	0	2 (7)
	Nonframeshift	2 (8)	0	2 (7)
	Synonymous	2 (8)	0	2 (7)
Digenic	Frameshift (*COL4A4*), missense (*COL4A3*)	0	1 (100)	1 (50)
	Nonsense (*COL4A4*), missense (*COL4A3* and *COL4A4*)	1 (100)	0	1 (50)
Autosomal recessive or dominant^a^	Missense	2 (67)	3 (60)	5 (63)
	Frameshift, missense	0	1 (20)	1 (13)
	Noncoding	1 (33)	0	1 (13)
	Splice site, missense	0	1 (200)	1 (13)
Unknown	Missense	2 (33)	0	2 (33)
	Missense, noncoding	1 (17)	0	1 (17)
	Missense, synonymous	1 (17)	0	1 (17)
	Nonframeshift, synonymous	1 (17)	0	1 (17)
	Noncoding, missense	1 (17)	0	1 (17)
No mutation identified	No mutation identified	5 (100)	2 (100)	7 (100)
Missing	Missing	5 (100)	4 (100)	9 (100)

ARAS, autosomal recessive Alport syndrome.

a Autosomal compound heterozygous patients were classified as “autosomal recessive or dominant,” if their biopsy was suggestive for ARAS; however, only 1 likely pathogenic or pathogenic variant plus 1 variant of unknown significance was identified.

The mean baseline eGFR was 64 ml/min (range: 18–124 ml/min). Mean proteinuria was 1844 mg/24 h. Mean blood pressure was 126/75 mmHg (Table 1). Body mass index showed a broad range from 17.8 to 51.1 kg/m^2^ (mean 26.76) (Table 1).

**Figure 2:**
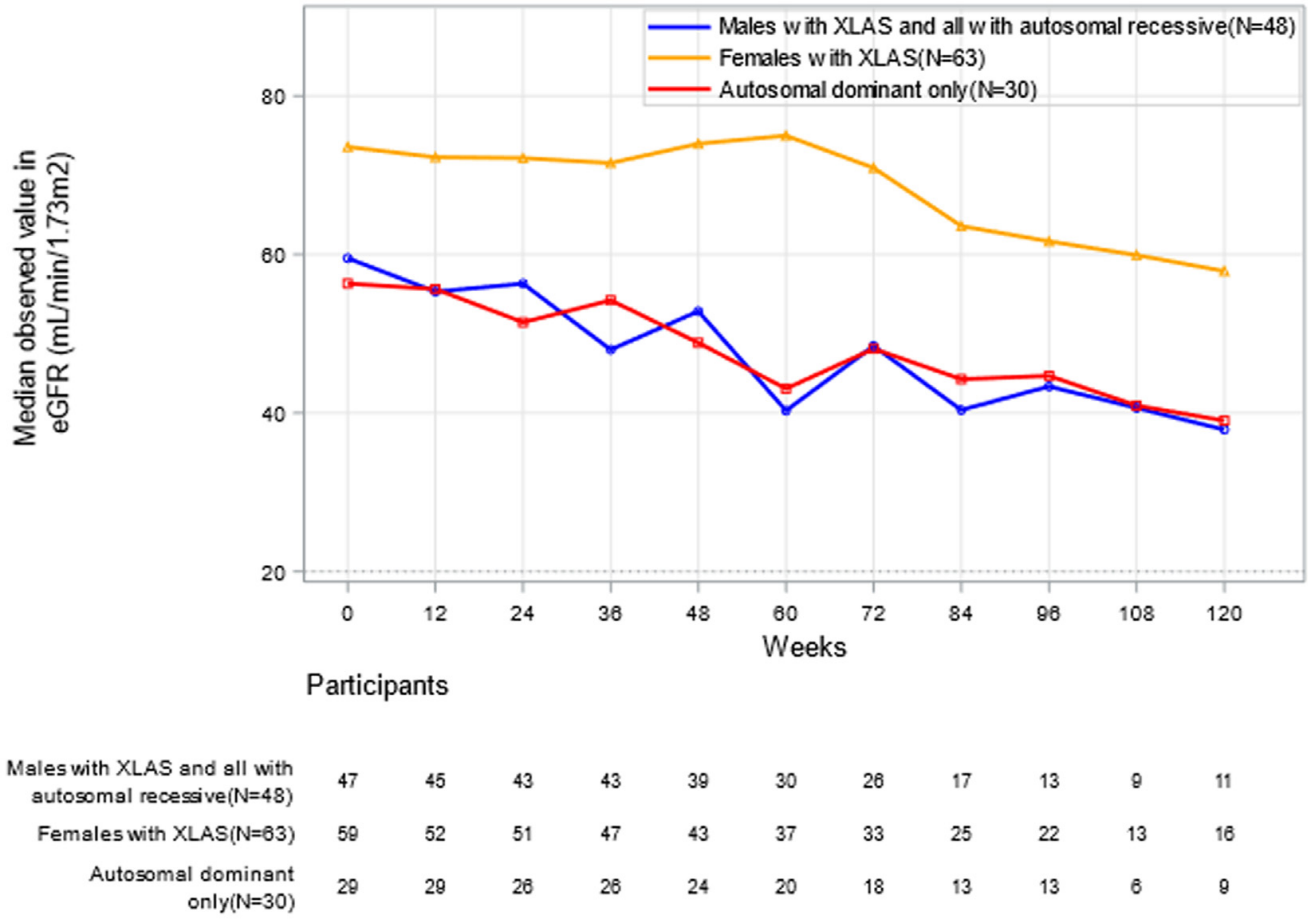
Median plot in eGFR_CKD-EPI_ (ml/min per 1.73 m^2^) over time by genotype subgroup in all enrolled participants. For the blue line, in addition to the 38 hemizygous XLAS males, for participants with autosomal inheritance, 10 were classified as ARAS (2 with homozygous ARAS and 8 with compound heterozygous ARAS with 1 variant classified as pathogenic or likely pathogenic and the other classified as “variant of unknown significance,” but clear morphologic picture of ARAS in the kidney biopsy). For the red line, the 30 individuals were analyzed and classified as autosomal dominant (ADAS) with only one pathogenic or likely pathogenic variant (excluding the 8 individuals, classified as ARAS by histology, see above). ADAS, autosomal dominant Alport syndrome; ARAS, autosomal recessive Alport syndrome; CKD-EPI, Chronic Kidney Disease Epidemiology Collaboration equation; eGFR, estimated glomerular filtration rate; XLAS, X-linked Alport syndrome.

In Figure 2, we display the median loss in eGFR over time. In the 42 patients who completed the full course of the study, mean eGFR was 59.0 ml/min per 1.73 m^2^ (*n* = 42, SD = 19.4) at baseline and 53.0 ml/min per 1.73 m^2^ (SD=25.4) at week 120 with a mean change of −6.0 ml/min per 1.73 m^2^ (SD = 13.6) resulting in an average annual change in eGFR of −2.6 ml/min per 1.73 m^2^ per year (Supplementary Table S2). The GFR trend among the 153 participants with at least 2 GFR measurements was −2.94 ml/min per 1.73 m^2^ per year (SD = 10.7 ml/min per 1.73 m^2^ per year).

The summary of eGFR change during the study by subgroup is provided in Tables 3 and 4, with a further breakdown by baseline UPCR groups provided in Supplementary Table S3. Among participants with X-linked *COL4A5* genotype, males had a mean eGFR slope of −6.7 ml/min per 1.73 m^2^ per year (SD = 8.3 ml/min per 1.73 m^2^ per year) versus 0.6 ml/min per 1.73 m^2^ per year (SD = 13.2 ml/min per 1.73 m^2^ per year) in females. Of the 28 subjects with autosomal dominant inheritance, the mean eGFR slope was−1.66 ml/min per 1.73 m^2^ per year (SD = 4.71 ml/min per 1.73 m^2^ per year) without sex differences; 5 males (18%) had a mean eGFR slope of −2.8 ml/min per 1.73 m^2^ per year (SD = 3.1 ml/min per 1.73 m^2^ per year) versus −1.4 ml/min per 1.73 m^2^ per year (SD = 5.01 ml/min per 1.73 m^2^ per year) in 23 (82%) females with a *P*-value of difference of 0.5712. The 2 subjects (both female) with autosomal recessive inheritance had an eGFR slope of −8.7 and −7.5 ml/min per 1.73 m^2^ per year.

**Table 3:** Summary of the key disease characteristics in urine assessments at baseline and eGFR slope_CKD-EPI_ (ml/min per 1.73 m^2^ per yr)

Variable	Statistics	Baseline UACR (mg/g)					
		> 1000 (*n* = 34)			≤ 1000 (*n* = 129)		
		Female (*n* = 11)	Male (*n* = 23)	Overall (*n* = 34)	Female (*n* = 97)	Male (*n* = 32)	Overall (*n* = 129)
eGFR slope (CKD-EPI 2009) (ml/min per 1.73 m^2^ per yr)	Mean (SD)	−6.68 (6.99)	−11.91 (11.36)	−10.16 (10.31)	−0.31 (10.96)	−2.58 (5.99)	−0.90 (9.93)
	Median	−4.68	−9.83	−8.66	−1.14	−2.37	−1.42
	Min; max	−18.7; 3.5	−45.0; 3.7	−45.0; 3.7	−20.5; 89.7^a^	−19.3; 16.0	−20.5; 89.7^a^
	*P*-value						<0.0001
Baseline eGFR value (CKD-EPI 2009) (ml/min per 1.73 m^2^ per yr)	Mean (SD)	50.7 (13.0)	54.8 (20.4)	53.4 (18.2)	68.6 (22.1)	60.9 (19.8)	66.6 (21.7)
	Median	49.9	53.2	51.3	69.6	59.6	65.5
	Min; max	31.3; 73.6	24.0; 86.2	24.0; 86.2	26.0; 124.4	17.8; 109.7	18; 124
Baseline UACR (mg/g)	Mean (SD)	1780 (970)	1940 (1,030)	1880 (1,000)	190 (240)	290 (280)	210 (250)
	Median	1250	1540	1510	90	210	110
	Min; max	1100; 4,200	1000; 5,800	1000; 5,800	0.0; 1000	0.0; 1000	0.0; 1000

CKD-EPI, Chronic Kidney Disease Epidemiology Collaboration equation; eGFR, estimated glomerular filtration rate; N1, number of participants with value; UACR, urine albumin-to-creatinine ratio.

a This upper range value (unusual positive eGFR slope) can be explained by an individual with eGFR values of 98, 133, and 140.7 ml/min per 1.73 m^2^ collected ondays 8, 75, and 173, respectively. Therefore, the slope was estimated at 89.7.

**Figure 3:**
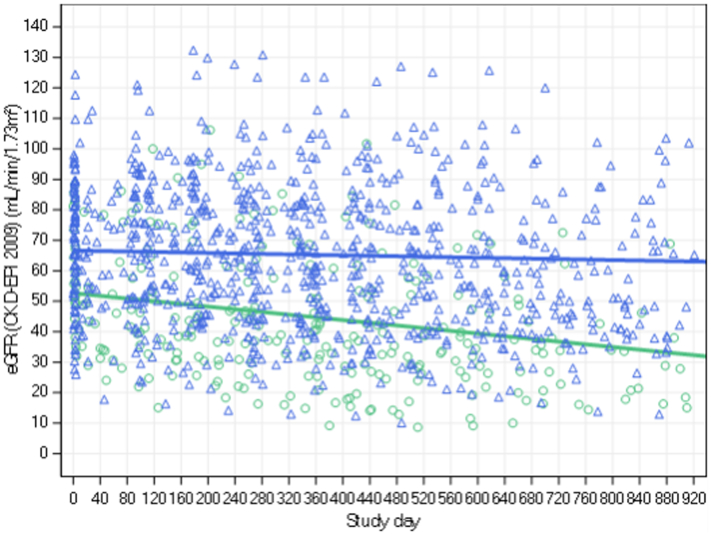
eGFR slope in participants with high versus low baseline UACR severity. Green line: participants with baseline UACR > 1 g/g (or UPCR > 2 g/g), *n* = 37.Blue line: participants with baseline UACR ≤ 1 g/g (and UPCR ≤ 2 g/g, or the assessment met the criterion when only 1 assessment was performed), *n* = 126. CKD-EPI, Chronic Kidney Disease Epidemiology Collaboration; eGFR, estimated glomerular filtration rate; UACR, urine albumin-to-creatinine ratio; UPCR, urine protein-to-creatinine ratio.

In Figure 3, we illustrate the eGFR slope in patients with high versus low baseline proteinuria severity. Severe baseline proteinuria (defined by UPCR > 2 g/g or UACR > 1000 mg/g) was significantly associated (*P* < 0.0001) with more rapid loss of eGFR (−10.16 ml/min per 1.73 m^2^ per year for UACR and −10.55 ml/min per 1.73 m^2^ per year for UPCR) versus nonsevere baseline proteinuria (eGFR slope of −0.90 ml/min per 1.73 m^2^ per year for UACR and −1.26 ml/min per 1.73 m^2^ per year for UPCR) (Table 3, Table 4 and Supplementary Table S3).

**Table 4:** Summary of the key disease characteristics in genotype at baseline and eGFR slope_CKD-EPI_ (ml/min per 1.73 m^2^per yr)

Variables	Statistics	Likely inherence							
		XLAS (*n* = 101)		ADAS (*n* = 30)		ARAS (*n* = 2)		Other^a^ (*n* = 32)	
		Female (*n* = 63)	Male (*n* = 38)	Female (*n* = 24)	Male (*n* = 6)	Female (*n* = 2)	Male (*n* = 0)	Female (*n* = 20)	Male (*n* = 12)
eGFR slope (CKD-EPI 2009) (ml/min per 1.73 m^2^ per yr)	Mean (SD)	0.61 (13.24)	−6.69 (8.25)	−1.42 (5.01)	−2.77 (3.14)	−8.06 (0.84)	NA	−4.81 (6.10)	−7.29 (14.58)
	Median	−0.38	−4.64	−1.45	−2.51	−8.06	NA	−3.34	−4.70
	Min; max	−20.5; 89.7^b^	−34.4; 6.6	−13.2; 9.3	−5.9; 1.4	−8.7; −7.5	NA	−18.7; 3.2	−45.0; 16.0
Baseline eGFR value (CKD-EPI 2009) (ml/min per 1.73 m^2^ per yr)	Mean (SD)	71.0 (21.5)	60.0 (21.6)	65.3 (22.9)	48.1 (18.3)	58.7 (13.4)	NA	57.4 (21.2)	60.7 (14.6)
	Median	73.60	59.65	66.57	51.38	58.70	NA	56.16	56.72
	Min; max	30.4; 124.4	24.0; 109.7	27.5; 109.7	17.8; 70.2	49.2; 68.2	NA	26.0; 112.6	42.3; 83.4
Baseline albumin-to-creatinine ratio (mg/g)	Mean (SD)	200 (300)	1060 (1160)	520 (900)	520 (490)	1650 (770)	NA	460 (700)	960 (1,010)
	Median	90	940	110	390	1650	NA	210	640
	Min; max	0.0; 1300	0.0; 5800	0.0; 4200	0.0; 1,400	1100; 2200	NA	0.0; 2800	0.0; 3,100
Age (yrs)	Mean (SD)	47.8 (13.3)	35.7 (13.3)	50.6 (10.5)	54.7 (16.0)	33.00 (0.00)	NA	48.4 (15.4)	37.0 (12.6)
	Median	49.0	33.0	49.0	57.0	33.0	NA	55.0	33.0
	Min; max	15.0; 80.0	17.0; 60.0	25.0; 69.0	30.0; 76.0	33.0; 33.0	NA	25.0; 68.0	19.0; 57.0

ADAS, autosomal dominant Alport syndrome; ARAS, autosomal recessive Alport syndrome; CKD-EPI, Chronic Kidney Disease Epidemiology Collaboration equation; eGFR, estimated glomerular filtration rate; NA, not applicable; XLAS, X-linked Alport syndrome.

a Other: when the likely inheritance is in “ARAS or ADAS,” or “Digenic,” or “unknown” or “missing.”

b This upper range value (unusual positive eGFR slope) can be explained by an individual with eGFR values of 98, 133, and 140.7 ml/min per 1.73 m^2^ collected on days 8, 75, and 173, respectively. Therefore, the slope in this individual was estimated at 89.7.

**Figure 4:**
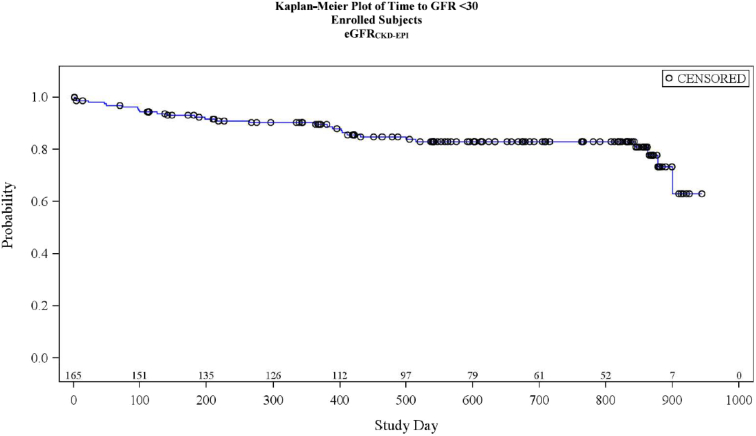
Kaplan–Meier plot of time to GFR < 30 ml/min per 1.73 m^2^ in all participants by eGFR_CKD-EPI_ equation. Time to GFR < 30 ml/min per 1.73 m^2^ was defined as the time from enrollment to the date of GFR < 30 ml/min per 1.73 m^2^. Participants who did not have GFR < 30 ml/min per 1.73 m^2^ were censored at their last GFR assessment. CKD-EPI, Chronic Kidney Disease Epidemiology Collaboration; eGFR, estimated glomerular filtration rate; GFR, glomerular filtration rate. Notes: Time to GFR < 30 ml/min per 1.73 m^2^ was defined as the time from enrollment (informed consent date) to the date of GFR< 30. Subjects who did not have GFR < 30 were censored at their last GFR assessment. Subjects enrolled in Amendment 4 of the protocol did not have measured GFR. Subjects enrolled prior to Amendment 3 of the protocol did not have cystatin collected.

The Kaplan–Meier plot of time to GFR < 30 ml/min per 1.73 m^2^ in all participants is shown in Figure 4. Of the 165 patients who participated in the study, 28 (17%) progressed to GFR < 30 ml/min per 1.73 m^2^ (Figure 4).

Among all potential predictors of rapid progression (defined as rapid loss of eGFR) discussed in the multivariable logistic regression *posthoc* analyses section (Supplemental Material), the baseline UPCR was the best predictor. The odds ratio of baseline UPCR (g/g) is 2.19 (confidence interval: 1.49–3.23; *P* = 0.0001) (Table 5). This odds ratio can be interpreted as every unit increase of UPCR increases the odds of being a rapid progressor by 2.19 folds.

**Table 5:** Odds ratio estimates for logistic regression on eGFRslopein rapid progressors versus nonrapid progressors

Variable	Rapid progressor (*n* = 54)	Nonrapid progressor (*n* = 99)	Odds ratio
eGFRslope (ml/min per 1.73 m^2^ per yr)			
Mean (SD)	−10.6 (7.7)	1.3 (9.7)	
Median	−7.8	−0.2	
Min; max	−45.0; −4.1	−4.0; 89.7^a^	
Baseline urine protein-to-creatinine ratio (g/g)			
Mean (SD)	1.83 (1.67)	0.73 (0.84)	
Median	1.29	0.32	
Min; max	0.1; 6.5	0.1; 4.3	
Estimated odds ratio (95% CI)			2.19 (1.49, 3.23)
*P*-value			0.0001
Sex, *n* (%)			
Male	29 (53.7)	25 (25.3)	
Female	25 (46.3)	74 (74.7)	

CI, confidence interval; CKD-EPI, Chronic Kidney Disease Epidemiology Collaboration equation; eGFR, estimated glomerular filtration rate.

The rapid progressor was defined when the patient's eGFR slope is ≤ −4ml/min per 1.73 m^2^ per yr during the study.

a This upper range value (unusual positive eGFR slope) can be explained by an individual with eGFR values of 98, 133, and 140.7 ml/min per 1.73 m^2^ collected on days 8, 75, and 173, respectively. Therefore, the slope in this individual was estimated at 89.7.

The Pearson correlation between eGFR slope and key urine biomarkers at baseline is provided in Supplementary Table S4. Negative linear correlations were observed between eGFR and various biomarkers (the parameters were ordered by correlation strength from strong to weak), including both serum and urine neutrophil gelatinase-associated lipocalin (*r* = −0.63 and −0.42, respectively, both *P* < 0.0001), urine cystatin C (*r* = −0.53, *P* < 0.0001), urine clusterin (*r* = 0.41, *P* < 0.0001), urine β-2-microglobulin (*r* = −0.4, *P* < 0.0001), urine kidney injury molecule-1 (*r* = −0.25, *P* < 0.0001), and asymmetric dimethylarginine (*r* = −0.21, *P* < 0.0001) (Supplementary Table S4).

The overall rates of AEs and SAEs between males and females (63% vs. 64% and 7% vs. 7%, respectively) were comparable (Table 6). There were no AE-related deaths during the study. More SAEs were reported in males than in females (13% vs. 4%, respectively), and 1 life-threatening AE (appendicitis perforated) was reported in a female participant. AEs related to AS (18% vs. 15%) and another medical condition (30% vs. 32%) were also similar in male versus female participants. One male participant experienced an AE (chronic kidney failure) that led to study termination. The rate of AEs was comparable in participants with baseline eGFR < 60 ml/min per 1.73 m^2^ to that in participants with eGFR ≥ 60 ml/min per 1.73 m^2^ (65% vs. 61%), as was the overall rate of SAEs (8% vs. 7%). However, more participants with eGFR < 60 ml/min per 1.73 m^2^ experienced AEs related to AS (27% vs. 7%). Sex differences in AEs included urinary tract infections (9.2% of females, none in males), gout (4.6% of females, 1.8% of males), and psychiatric disorders (6.4% of females, 3.6% of males). Fatigue was more common in males (0.9% of females, 8.9% of males); however, hypotension was more common in females (3.7% of females, none in males) as was hypertension (2.8% of females, none in males). High potassium levels were reported in 7.1% of males, but not in females. Acute kidney failure was reported in 3.6% of males, but not in females.

**Table 6:** Overall summary of adverse events

Adverse events	Female (*n* = 109)	Male (*n* = 56)	Overall (*n* = 165)	eGFR ≥ 60 (*n* = 85)	eGFR< 60 (*n* = 75)
AEs total number^a^	245	108	353	159	182 (53.4%)
Mild	29 (26.6%)	14 (25.0%)	43 (26.1%)	23 (27.1%)	18 (24.0%)
Moderate	36 (33.0%)	14 (25.0%)	50 (30.3%)	24 (28.2%)	24 (32.0%)
Severe	4 (3.7%)	7 (12.5%)	11 (6.7%)	4 (4.7%)	7 (9.3%)
Life-threatening	1 (0.9%)	0 (0.0%)	1 (0.6%)	1 (1.2%)	0 (0.0%)
Death	0 (0.0%)	0 (0.0%)	0 (0.0%)	0 (0.0%)	0 (0.0%)
AEs leading to study termination	0 (0.0%)	1 (1.8%)	1 (0.6%)	0 (0.0%)	1 (1.3%)
SAEs	8 (7.3%)	4 (7.1%)	12 (7.3%)	6 (7.1%)	6 (8.0%)
Causal relationship^b^					
Study-related procedure	1 (0.9%)	0 (0.0%)	1 (0.6%)	1 (1.2%)	0 (0.0%)
Alport syndrome	16 (14.7%)	10 (17.9%)	26 (15.8%)	6 (7.1%)	20 (26.7%)
Other medical condition	35 (32.1%)	17 (30.4%)	52 (31.5%)	24 (28.2%)	26 (34.7%)
Other	54 (49.5%)	20 (35.7%)	74 (44.8%)	38 (44.7%)	38 (44.7%)
AEs by system organ class^c^^,^^d^	70 (64.2%)	35 (62.5%)	105 (63.6%)		
Infections and infestations	41 (37.6%)	16 (28.6%)	57 (34.5%)		
Upper respiratory tract infection	9 (8.3%)	5 (8.9%)	14 (8.5%)		
Nasopharyngitis	7 (6.4%)	4 (7.1%)	11 (6.7%)		
Urinary tract infections	10 (9.2%)	0 (0.0%)	10 (6.1%)		
Sinusitis	7 (6.4%)	1 (1.8%)	8 (4.8%)		
Ear infection	2 (1.8%)	4 (7.1%)	6 (3.6%)		
Bronchitis	3 (2.8%)	1 (1.8%)	4 (2.4%)		
Influenza	4 (3.7%)	0 (0.0%)	4 (2.4%)		
Pneumonia	3 (2.8%)	0 (0.0%)	3 (1.8%)		
Appendicitis	1 (0.9%)	1 (1.8%)	2 (1.2%)		
Fungal infection	1 (0.9%)	1 (1.8%)	2 (1.2%)		
Viral infection	1 (0.9%)	1 (1.8%)	2 (1.2%)		
Musculoskeletal and connective tissue disorders	19 (17.4%)	7 (12.5%)	26 (15.8%)		
Back pain	6 (5.5%)	0 (0.0%)	6 (3.6%)		
Arthralgia	3 (2.8%)	1 (1.8%)	4 (2.4%)		
Flank pain	1 (0.9%)	2 (3.6%)	3 (1.8%)		
Muscle spasms	1 (0.9%)	2 (3.6%)	3 (1.8%)		
Musculoskeletal pain	1 (0.9%)	2 (3.6%)	3 (1.8%)		
Pain in extremity	1 (0.9%)	1 (1.8%)	2 (1.2%)		
Tendonitis	2 (1.8%)	0 (0.0%)	2 (1.2%)		
Gastrointestinal disorders	14 (12.8%)	5 (8.9%)	19 (11.5%)		
Gastroesophageal reflux disease	4 (3.7%)	1 (1.8%)	5 (3.0%)		
Diarrhea	1 (0.9%)	3 (5.4%)	4 (2.4%)		
Nausea	2 (1.8%)	1 (1.8%)	3 (1.8%)		
Dyspepsia	2 (1.8%)	0 (0.0%)	2 (1.2%)		
Toothache	2 (1.8%)	0 (0.0%)	2 (1.2%)		
Vomiting	2 (1.8%)	0 (0.0%)	2 (1.2%)		
General disorders and administration site conditions	10 (9.2%)	8 (14.3%)	18 (10.9%)		
Fatigue	1 (0.9%)	5 (8.9%)	6 (3.6%)		
Chest pain	2 (1.8%)	0 (0.0%)	2 (1.2%)		
Influenza like illness	1 (0.9%)	1 (1.8%)	2 (1.2%)		
Edema peripheral	2 (1.8%)	0 (0.0%)	2 (1.2%)		
Pyrexia	1 (0.9%)	1 (1.8%)	2 (1.2%)		
Metabolism and nutrition disorders	10 (9.2%)	6 (10.7%)	16 (10.9%)		
Gout	5 (4.6%)	1 (1.8 %)	6 (3.6%)		
Hyperkalemia	0 (0.0%)	4 (7.1%)	4 (2.4%)		
Hyperlipidemia	3 (2.8%)	1 (1.8%)	4 (2.4%)		
Hypokalemia	2 (1.8%)	0 (0.0%)	2 (1.2%)		
Nervous system disorders	9 (8.3%)	4 (7.1%)	13 (7.9%)		
Headache	6 (5.5%)	2 (3.6%)	8 (4.8%)		
Dizziness	1 (0.9%)	2 (3.6%)	3 (1.8%)		
Investigations	8 (7.3%)	4 (7.1%)	12 (7.3%)		
Blood creatinine increased	2 (1.8%)	2 (3.6%)	4 (2.4%)		
Smear cervix abnormal	2 (1.8%)	0 (0.0%)	2 (1.2%)		
Vitamin D decreased	1 (0.9%)	1 (1.8%)	2 (1.2%)		
Respiratory, thoracic and mediastinal disorders	9 (8.3%)	2 (3.6%)	11 (6.7%)		
Cough	4 (3.7%)	1 (1.8%)	5 (3.0%)		
Ear and labyrinth disorders	9 (8.3%)	1 (1.8%)	10 (6.1%)		
Deafness	4 (3.7%)	1 (1.8%)	4 (2.4%)		
Vertigo	2 (1.8%)	0 (0.0%)	2 (1.2%)		
Injury, poisoning and procedural complications	6 (5.5%)	3 (5.4%)	9 (5.5%)		
Muscle strain	2 (1.8%)	0 (0.0%)	2 (1.2%)		
Psychiatric disorders	7 (6.4%)	2 (3.6%)	9 (5.5%)		
Anxiety	2 (1.8%)	1 (1.8%)	3 (1.8%)		
Stress	2 (1.8%)	0 (0.0%)	2 (1.2%)		
Skin and subcutaneous tissue disorders	7 (6.4%)	2 (3.6%)	9 (5.5%)		
Dermatitis contact	2 (1.8%)	0 (0.0%)	2 (1.2%)		
Cardiac disorders	6 (5.5%)	2 (3.6%)	8 (4.8%)		
Palpations	2 (2.8%)	0 (0.0%)	3 (1.8%)		
Immune system disorders	3 (2.8%)	4 (7.1%)	7 (4.2%)		
Hypersensitivity	3 (2.8%)	2 (3.6%)	5 (3.0%)		
Seasonal allergy	0 (0.0%)	2 (3.6%)	2 (1.2%)		
Surgical and medical procedures	5 (4.6%)	2 (3.6%)	7 (4.2%)		
Vascular disorders	7 (6.4%)	0 (0.0%)	7 (4.2%)		
Hypotension	4 (3.7%)	0 (0.0%)	4 (2.4%)		
Hypertension	3 (2.8%)	0 (0.0%)	3 (1.8%)		
Blood and lymphatic system disorders	2 (1.8%)	4 (7.1%)	6 (3.6%)		
Anemia	2 (1.8%)	3 (5.4%)	5 (3.0%)		
Renal and urinary disorders	3 (2.8%)	3 (5.4%)	6 (3.6%)		
Renal failure acute	0 (0.0%)	2 (3.6%)	2 (1.2%)		
Reproductive system and breast disorders	3 (2.8%)	1 (1.8%)	4 (2.4%)		
Eye disorders	2 (1.8%)	1 (1.8%)	3 (1.8%)		
Hepatobiliary disorders	2 (1.8%)	0 (0.0%)	2 (1.2%)		
Endocrine disorders	1 (0.9%)	0 (0.0%)	1 (0.6%)		
Pregnancy, puerperium and perinatal conditions	1 (0.9%)	0 (0.0%)	1 (0.6%)		

AE, adverse event; SAE, serious AE.

a Subjects reporting more than one adverse event are counted only once using the highest severity.

b Subjects may be counted in each relationship category. Subjects reporting more than one adverse event within each relationship category are counted only once.

c Adverse events are coded to system organ class and preferred term using the Medical Dictionary for Regulatory Activities (MedDRA), version 17.0.

d Only events affecting > 1% of the subjects are shown.

## Discussion

ATHENA is the first large scale prospective natural history study of patients with AS using ICH-GCP standards of data collection, including an AE and SAE reporting system. The data generated by this study can be used for stratification of participants and establishment of endpoints for interventional trials in AS. A strength of this study is that over 80% of participants received RAS blockade, the current standard of care for Alport kidney disease.^13^^,^^19^ Results of this study demonstrated that baseline UPCR and UACR, along with genotype, were the best predictors of eGFR decline.

Our study was purely observational and noninterventional, so we considered that some of our patients may have lacked motivation to complete the study because the study did not include a new, potentially effective drug despite their ongoing progressive kidney disease. Still, only 17.0% (*n* = 28) of ATHENA patients dropped out prematurely for reasons other than “study termination by sponsor.”

In line with previous studies, most participants in this study had variants in *COL4A5*,^1^ followed by autosomal dominant AS, and a small proportion of participants had autosomal recessive disease. The results demonstrate that many patients with A S have rapidly declining kidney function, defined as a decline of ≥ 4 ml/min per 1.73 m^2^. Similar to a previous retrospective study,^3^ a high number of patients with low GFR and high albuminuria at enrollment showed a decline of their eGFR despite RAAS therapy. Risk factors for rapid progression included a UPCR > 2 g/g, UACR > 1000 mg/g, or male patients aged 12 to 23 years with eGFR < 90 ml/min per 1.73 m^2^.

Previous studies showed that male hemizygous patients with XLAS have a 100% estimated risk of ESKF, whereas heterozygous female patients with XLAS have an estimated risk of ESKF of up to 40%, which can be reduced by timely RAAS blockade.^1^^,^^8^ Patients with autosomal dominant disease have a 20% estimated risk of ESKF.^20^ The results of our current study are supportive of these phenotypic differences. We demonstrated that more males than females, more participants with lower baseline eGFR (< 60 ml/min per 1.73 m^2^), and male participants with X-linked disease were more likely to reach eGFR levels< 30 ml/min per 1.73 m^2^ over the study period.

AS has been more commonly described in males, whereas most participants included in this study were female.^1^ Symptoms of AS tend to manifest at a very young age in male patients, which may mean that by the time adulthood was reached, prospective male participants did not meet the inclusion criteria for the ATHENA study because their eGFR may have been lower at a younger age than female patients. Previously, kidney involvement was shown to be widely variable in heterozygous women with AS, and potentially less severe than the disease in some male patients^1^^,^^13^; and here we show that there was more variability in kidney involvement in female patients than males, and female patients had less drastic eGFR slopes than males, indicating less severe kidney involvement or a better response to RAS blockade therapy. It must also be acknowledged that study participants with autosomal dominant disease represented a severe disease subset of the larger group of participants with heterozygous variants in *COL4A3* and *COL4A4*.

There are several limitations to this study. Only a small group of participants completed follow-up to 120 weeks such that the observation period was limited. The varied study length between subgroups may also result in bias, especially because overall participant numbers were low. Protocol amendments also meant that not all participants had iohexol-measured GFR, and not all had cystatin C data collected, which may make comparisons difficult. However, there is limited research on potential biomarkers of kidney progression in Alport disease. To our knowledge, this is the first study to show that albuminuria is a very strong biomarker of kidney progression in Alport disease. We also demonstrate that UACR correlates best with the decline of eGFR, but markers of tubular damage such as neutrophil gelatinase-associated lipocalin, kidney injury molecule-1, and clusterin also correlate with eGFR decline.

Similar to the EARLY PRO-TECT Alport, the CARDINAL and the HERA trial,^6^^,^^21^^,^^22^ the ATHENA trial collected data on AEs and SAEs under ICH-GCP standards. Considering that 80% of the patients were treated with RAS-blockade, the ATHENA study provides a unique source for future randomized, placebo-controlled trials such as DOUBLE PRO-TECT Alport,^23^ indicating which “natural” AEs can be expected under RAS-blockade as standard of care.^24^^,^^25^ The duration of patients’ therapy added up to a total of 216.4 patient-years on ramipril in the EARLY PRO-TECT trial, compared with 237.95 patient-years on RAS-blockade in the ATHENA trial. RAS-blockade in the ATHENA trial was well-tolerated in most patients with only 17 of 353 (4.5%) AEs, which could be associated with RAS-blockade (fatigue, hyperkalemia, hypotension, acute kidney injury, Table 6), similar to the normotensive children in the EARLY PRO-TECT Alport trial, in which up-titration of ramipril was well-tolerated with high maximum dosages of ramipril not different to placebo. In the EARLY PRO-TECT Alport trial, analysis of 465 AEs before disease progression revealed that ramipril therapy was safe in children. Similarly, the number of AEs and SAEs in the ATHENA trial did not differ significantly between patients treated with RAS-blockade and untreated patients.

In summary, this study provides data on rates of eGFR decline in patients with AS that should be useful in the design of interventional trials. Our data on “natural disease-related” common AEs and possible SAEs in patients with AS also should be useful in the design and interpretation of future randomized controlled trials. The ATHENA trial will help to interpret potential side effects of upcoming new therapies. For the first time, the study showed that UACR and UPCR correlate best with eGFR decline and identified other potential biomarkers that may serve as predictors of eGFR decline in future interventional clinical trials.

## Disclosure

OG (or his employer) received consulting fees from Bayer, Otsuka, Regulus, Sanofi, and 10.13039/100004325AstraZeneca. MNR received research grants and consulting honoraria from Chinook, Kaneka, Otsuka, Reata Pharmaceuticals, Regulus, Sanofi, and Travere Therapeutics. JS received consulting fees from Reata Pharmaceuticals. QZ is employed by Sanofi and holds Sanofi stock. AH received consulting fees from Avidity and OAK Hill Bio and holds Sanofi stocks. JL is employed by Sanofi and holds Sanofi stock. SL is employed by Sanofi and holds Sanofi stock. CEK received support for the present manuscript (e.g., funding, provision of study materials, medical writing, article processing charges, etc.) and grants or contracts from Regulus and Sanofi.

## Supplementary Material

Supplementary File (PDF) **Supplementary Methods. Table S1.** Summary of the American College of Medical Genetics and Genomics classification of variants for all enrolled participants. **Table S2.** Estimated glomerular filtration rate change from baseline at week 120 in those individuals, who completed the full 120 weeks of the study. **Table S3.** Summary of the key baseline characteristics during the study broken down by baseline urine protein-to-creatinine ratio. **Table S4.** Summary of the partial correlation between eGFR (ml/min per 1.73 m^2^) and biomarkers over time during the study.

## Footnotes

Supplementary File (PDF)

**Supplementary Methods.**
**Table S1.** Summary of the American College of Medical Genetics and Genomics classification of variants for all enrolled participants. **Table S2.** Estimated glomerular filtration rate change from baseline at week 120 in those individuals, who completed the full 120 weeks of the study. **Table S3.** Summary of the key baseline characteristics during the study broken down by baseline urine protein-to-creatinine ratio. **Table S4.** Summary of the partial correlation between eGFR (ml/min per 1.73 m^2^) and biomarkers over time during the study.
