# Supplementary material for: Prospective Cohort Study in Alport Syndrome Patients Under Standard Therapy
Source: Kidney Int Rep. 2025 Mar 6;10(5):1360–71. doi: 10.1016/j.ekir.2025.02.036 (PMC12142643; doi:10.1016/j.ekir.2025.02.036)
Supplement: Supplementary File (PDF) — Supplementary Methods. Table S1. Summary of the American College of Medical Genetics and Genomics classification of variants for all enrolled participants. Table S2. Estimated glomerular filtration rate change from baseline at week 120 in those individuals, who completed the full 120 weeks of the study. Table S3. Summary of the key baseline characteristics during the study broken down by baseline urine protein-to-creatinine ratio. Table S4. Summary of the partial correlation between eGFR (ml/min per 1.73 m2) and biomarkers over time during the study. [file mmc1.pdf]

**<u>Supplemental materials</u>**

## Supplemental methods

### *Inclusion criteria*

Key inclusion criteria were a confirmed diagnosis of Alport syndrome (clinical, histopathologic, and/or genetic diagnosis), age 16–≤65 years (at study onset), the ability to understand and comply with the study requirements and provide written informed consent (assent for pediatric patients), and directly measured iohexol GFR 30–75mL/min/1.73m$^2$ within 30 days of enrollment (Delanaye, 2016). Subsequent protocol amendments changed the eligible age to 12–65 years and the GFR calculation method to the eGFR using the Chronic Kidney Disease Epidemiology Collaboration (CKD-EPI) creatinine-cystatin C equation, with a new range of 45–90mL/min/1.73m$^2$ within 30 days of enrollment for participants aged ≥16 years. For participants aged ≤15 years, eGFR was calculated using the Chronic Kidney Disease in Children (CKiD) equation (Pierce, 2021). Participants who met the following criteria were excluded from the study: use of investigational drugs at time of enrollment, or within 30 days, or five half-lives of enrollment, whichever was longer; and ongoing chronic hemodialysis therapy and/or kidney transplant.

### *Genetic sequencing*

The three genes *COL4A3, COL4A4,* and *COL4A5* were sequenced using a next-generation amplicon sequencing technique at Machaon Diagnostics (Berkeley, CA). All bases of the exons, plus 20 bases of flanking intronic sequence, and specific non-coding regions known to include pathogenic variants were sequenced. The DNA sequences were compared to the reference Human genome (Hg19) sequence. Variants were classified according to guidelines from the American College of Medical Genetics and Genomics and the Association for Molecular Pathology (1,2). Copy number of the *COL4A5* gene was determined using real-time polymerase chain reaction.

### *Urine and blood biomarker assessments*

Exploratory urine (β-2-microglobulin, clusterin, cystatin C, KIM-1, and NGAL) and blood (asymmetric dimethylarginine [ADMA], connective tissue growth factor [CTGF], neutrophil gelatinase-associated lipocalin [NGAL], and transforming growth factor β1 [TGF-β1]) biomarkers were assessed at baseline, every 24 weeks through to Week 96, and at the end of study or early-termination visit at Week 120. These included creatinine clearance, fractional excretion of sodium, total protein (including microRNA) and total albumin, blood urea nitrogen, serum creatinine (for eGFR), total bilirubin, alanine aminotransferase (ALT), aspartate aminotransferase (AST), alkaline phosphatase, serum cystatin C (for estimated glomerular filtration rate [eGFR]), sodium, potassium, calcium, glucose, chloride, bicarbonate, and lipid panel.

Further *post-hoc* analyses included using multivariable logistic regression to assess the odds ratio of rapid eGFR progression, defined as eGFR slope ≤−4mL/min/1.73m$^2$/year. Covariates were added to the model using forward selection and included: sex (male or female); age in years (age at the date of informed consent); race (White or not); baseline urine proteinuria (urine protein/creatinine ratio [UPCR]

≥2g/g or urine albumin/creatinine ratio [UACR] ≥1,000mg/g, or neither UPCR ≥2g/g nor UACR ≥1,000mg/g); baseline UPCR (g/g), baseline UACR (mg/g); baseline eGFR (eGFR <60mL/min/1.73m$^2$ or ≥60mL/min/1.73m$^2$, eGFR <90mL/min/1.73m$^2$ or ≥90mL/min/1.73m$^2$); baseline systolic blood pressure (SBP) (<130mmHg or ≥130mmHg); baseline diastolic blood pressure (DBP) (<80mmHg or ≥80mmHg); gene with variant (participant with *COL4A5* or not); likely mode of inheritance (X-linked or not) for those without variants in *COL4A5*; and biomarkers at baseline including β-2 macroglobulin, clusterin, cystatin C, and NGAL from both serum and urine. Variables found to be significantly associated with rapid eGFR progression (nominal p-value <0.05) were included as covariates in the final model. Secondary analyses included a time-to-event analysis as well as correlation analysis between longitudinal eGFR and longitudinal biomarker assessments. Regarding time-to-event analyses, an event was defined as GFR <30mL/min/1.73m$^2$, and time to event was defined as the time from enrollment (informed consent date) to the date of GFR <30mL/min/1.73m$^2$. Subjects who did not have GFR <30mL/min/1.73m$^2$ were censored at their last GFR assessment. For correlation analysis, partial correlation coefficient was calculated while adjusting for the repeated measures within the participant.

**Supplemental Table S1.** Summary of ACMG classification of variants for all enrolled participants

| Variant of highest pathogenicity, n (%) | Gene with variant | | | | | | | |
|---|---|---|---|---|---|---|---|---|
| | ***COL4A3* (N=17)** | ***COL4A4* (N=23)** | ***COL4A5* (N=101)** | ***COL4A3, COL4A4* (N=2)** | ***COL4A4, COL4A5* (N=5)** | ***COL4A3, COL4A4, COL4A5* (N=1)** | **No mutation identified (N=7)** | **Missing (N=9)** |
| Pathogenic | 8 (47.1) | 12 (52.2) | 63 (62.4) | 2 (100) | 0 | 0 | 0 | 0 |
| Likely pathogenic | 4 (23.5) | 6 (26.1) | 30 (29.7) | 0 | 0 | 0 | 0 | 0 |
| Uncertain significance | 5 (29.4) | 5 (21.7) | 8 (7.9) | 0 | 5 (100) | 1 (100) | 0 | 0 |
| Likely benign | 0 | 0 | 0 | 0 | 0 | 0 | 0 | 1 (11.1) |
| Benign | 0 | 0 | 0 | 0 | 0 | 0 | 0 | 2 (22.2) |
| No mutation identified | 0 | 0 | 0 | 0 | 0 | 0 | 7 (100) | 0 |
| Missing | 0 | 0 | 0 | 0 | 0 | 0 | 0 | 6 (66.7) |

N, total number of participants with each gene variant; n, number of participants with each pathogenicity.
ACMG, American College of Medical Genetics and Genomics; eGFR, estimated glomerular filtration rate.

**Supplemental Table S2.** eGFR change from baseline at Week 120 in those individuals, who completed the full 120 weeks of the study.

| Parameter | Visit | Observed value | Change from Baseline |
|---|---|---|---|
| $eGFR_{CKD\text{-}EPI\ creatinine}$ (mL/min/1.73m$^2$) | Baseline (N=42) | | |
| | Mean (SD) | 59.0 (19.4) | - |
| | Median (range) | 54.9 (24.0-102.0) | - |
| | Week 120 (N=42) | | |
| | Mean (SD) | 53.0 (25.4) | −6.0 (13.6) |
| | Median (range) | 47.7 (13.0-103.5) | -6.3 (-39.4-22.9) |

CKD-EPI, Chronic Kidney Disease Epidemiology Collaboration; eGFR, estimated glomerular filtration rate; SD, standard deviation.

**Supplemental Table S3.** Summary of the key baseline characteristics during the study broken down by baseline urine protein/creatinine

| Variable | Statistics | Baseline protein/creatinine ratio (g/g) | | | | | | | | | | | |
|---|---|---|---|---|---|---|---|---|---|---|---|---|---|
| | | ≤0.5 (N=75) | | | >0.5–1g/g (N=23) | | | >1–2g/g (N=27) | | | >2g/g (N=29) | | |
| | | Female (N1=59) | Male (N1=16) | Overall (N1=75) | Female (N1=15) | Male (N1=8) | Overall (N1=23) | Female (N1=15) | Male (N1=12) | Overall (N1=27) | Female (N1=10) | Male (N1=19) | Overall (N1=29) |
| | | | | | | | | | | | | | |

| | | | | | | | | | | | | | |
|---|---|---|---|---|---|---|---|---|---|---|---|---|---|
| **eGFR slope (CKD-EPI 2009) (mL/min/ 1.73m$^2$/ year)** | Mean (SD) | 1.23 (13.38) | −1.18 (5.70) | 0.71 (12.13) | −4.03 (4.76) | −5.40 (6.62) | −4.53 (5.39) | −2.20 (2.14) | −5.55 (5.37) | −3.81 (4.29) | −7.27 (8.11) | −12.37 (12.60) | −10.55 (11.32) |
| | Median | −0.18 | −1.50 | −0.88 | −3.54 | −2.71 | −3.42 | −2.64 | −6.58 | −3.45 | −8.06 | −9.71 | −8.95 |
| | Min; max | −20.5; 89.7* | −7.5; 16.0 | −20.5; 89.7* | −15.4; 2.4 | −19.3; 0.9 | −19.3; 2.4 | −5.1; 2.4 | −12.9; 6.6 | −12.9; 6.6 | −18.7; 3.5 | −45.0; 3.7 | −45.0; 3.7 |
| **Baseline eGFR value (CKD-EPI 2009) (mL/min/ 1.73m$^2$/ year)** | | | | | | | | | | | | | |
| | Mean (SD) | 70.6 (22.2) | 63.4 (20.4) | 69.1 (21.9) | 59.1 (17.4) | 67.7 (15.4) | 62.1 (16.9) | 64.6 (25.4) | 57.4 (23.2) | 61.3 (24.2) | 52.2 (16.9) | 51.1 (18.3) | 51.5 (17.5) |
| | Median | 70.9 | 58.2 | 69.5 | 56.6 | 70.0 | 64.4 | 70.1 | 59.6 | 62.5 | 50.3 | 46.5 | 49.2 |
| | Min; max | 26.0; 124.4 | 34.1; 109.7 | 26.0; 124.4 | 30.3; 85.6 | 47.2; 89.5 | 30.3; 89.5 | 27.5; 112.6 | 17.8; 86.2 | 17.8; 112.6 | 31.3; 88.2 | 24.0; 85.6 | 24.0; 88.2 |
| **Baseline albumin/ creatinine ratio (mg/g)** | | | | | | | | | | | | | |
| | Mean (SD) | 73.8 (91.4) | 111 (129) | 81.7 (101) | 319 (257) | 432 (313) | 355 (274) | 714 (293) | 923 (572) | 807 (442) | 1,656 (1,158) | 1,991 (1,136) | 1,875 (1,134) |
| | Median | 30.95 | 52.5 | 0.03509 | 0.262 | 0.352 | 0.286 | 0.679 | 0.876 | 0.691 | 1.221 | 1.830 | 1.540 |
| | Min; max | 3.9; 404 | 4.8; 425.8 | 3.9; 426 | 18.7; 1,183 | 85.4; 1,028 | 18.7; 1,183 | 27.6; 1,499 | 207.4; 2,234 | 207.4; 2,234 | 227; 4,189 | 445; 5,751 | 227; 5,751 |

* this upper range value (unusual positive eGFR slope) can be explained by one individual with eGFR values of 98, 133, and 140.7 ml/min/1.73m$^2$ collected at day 8, 75, and 173. Therefore the slope in this individual is estimated at 89.7.

Participants without baseline urine protein/creatinine assessment are not considered

CKD-EPI, Chronic Kidney Disease Epidemiology Collaboration; eGFR, estimated glomerular filtration rate; N1, number of participants with value; SD, standard deviation

**Supplemental Table S4.** Summary of the partial correlation between eGFR (mL/min/1.73m$^2$) and biomarkers over time during the study

| Parameter | Partial correlation coefficient | p-value |
|---|---|---|
| NGAL, serum (ng/mL) | −0.63 | <0.001 |
| Cystatin C, urine (ng/mg Cr), log transformed | −0.53 | <0.001 |
| NGAL, urine (ng/mg Cr), log transformed | −0.42 | <0.001 |
| Clusterin, urine (ng/mg Cr), log transformed | −0.41 | <0.001 |
| β-2 Microglobulin, urine (ng/mg Cr), log transformed | −0.40 | <0.001 |
| KIM-1, urine (pg/mg Cr), log transformed | −0.25 | <0.001 |
| ADMA (μmol/μmol Cr), log transformed | −0.21 | <0.001 |
| TGF- β1, urine (pg/mg Cr), log transformed | −0.05 | 0.3188 |
| CTGF, serum (ng/mL), log transformed | 0.09 | 0.0878 |

The hypothesis is to test whether the partial correlation coefficient is 0.
The partial correlation is adjusted at the participant level.
ADMA, asymmetric dimethylarginine; Cr, creatinine; CTGF, connective tissue growth factor; eGFR, estimated glomerular filtration rate; NGAL, neutrophil gelatinase-associated lipocalin; TGF-β1-transforming growth factor β1.
